# Multispectral Holographic Intensity and Phase Imaging of Semitransparent Ultrathin Films

**DOI:** 10.1021/acsphotonics.3c01834

**Published:** 2024-04-30

**Authors:** Sebastian Haegele, Daniel Martínez-Cercós, Javier Arrés Chillón, Bruno Paulillo, Roland A. Terborg, Valerio Pruneri

**Affiliations:** † ICFO-Institut de Ciències Fotòniques, The Barcelona Institute of Science and Technology, Castelldefels, 08860 Barcelona, Spain; ‡ ICREA-Institució Catalana de Recerca i Estudis Avançats, 08010 Barcelona, Spain

**Keywords:** microscopy, holography, interferometry, thin films, gold, dielectric constant

## Abstract

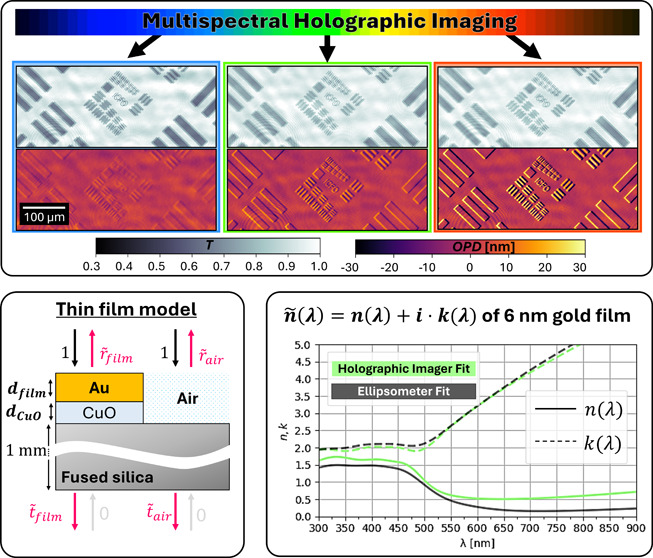

In this paper, we demonstrate a novel optical characterization method for ultrathin semitransparent and absorbing materials through multispectral intensity and phase imaging. The method is based on a lateral-shearing interferometric microscopy (LIM) technique, where phase-shifting allows extraction of both the intensity and the phase of transmitted optical fields. To demonstrate the performance in characterizing semitransparent thin films, we fabricated and measured cupric oxide (CuO) seeded gold ultrathin metal films (UTMFs) with mass-equivalent thicknesses from 2 to 27 nm on fused silica substrates. The optical properties were modeled using multilayer thin film interference and a parametric model of their complex refractive indices. The UTMF samples were imaged in the spectral range from 475 to 750 nm using the proposed LIM technique, and the model parameters were fitted to the measured data in order to determine the respective complex refractive indices for varying thicknesses. Overall, by using the combined intensity and phase not only for imaging and quality control but also for determining the material properties, such as complex refractive indices, this technique demonstrates a high potential for the characterization of the optical properties, of (semi-) transparent thin films.

## Introduction

Semitransparent thin films have become increasingly relevant in the fields of photonics and optoelectronics. Despite being only a few nanometers thick, the films can exhibit strong interactions with light, leading to a wide range of optical and optoelectronic applications. These include optical coatings,^1^ color filters,^2−5^ infrared absorbers,^6^ transparent conductors^7−11^ for displays or solar cells, meta-materials,^12−16^ meta-surfaces,^17,18^ and plasmonics,^19−22^ to name a few. An ongoing trend in photonics is the transition from thin to ultrathin films and atomically thin materials.^23^ In this paper, special attention has also been given to ultrathin metal films (UTMFs), as these show strong potential for use in optoelectronic devices due to their high conductivity^7^ and transparency,^9^ and also their tunable properties.^24−26^

The optical response of thin films depends on their thickness, morphology, and complex refractive index (RI). They can, for example, be modeled by multilayer interference, giving complex Fresnel coefficients with both intensity and phase, where the former describes the transmission, reflection, and absorption, while the latter refers to the refractive and diffractive characteristics of the films. The multilayer interference model, commonly implemented using the “transfer matrix method” (TMM),^27^ needs to include all dielectric and metallic layers in the calculations, together with their respective complex refractive indices and thicknesses, including the surrounding media and substrate itself. More specifically, the complex refractive index *ñ* = *n* + *i k* (with imaginary unit *i*) has a real part *n*, which is the refractive index that determines refraction and propagation speed, and an imaginary part *k*, which is the optical extinction coefficient. It is directly related to the complex dielectric constant *ε̃*_r_ of a (nonmagnetic) material via the relationship . It has been shown that the morphology of UTMFs depends on thickness,^24,25,28−33^ the deposition technique used,^34^ and the type of substrate and its pretreatment,^24,25,31^ and, therefore, the *ñ* of the films also depends on all of these parameters. When modeling the optical response of ultrathin films assuming a specific *ñ*, e.g., that of bulk material such as bulk gold or a thick gold film,^34−36^ this can lead to critical discrepancies between the modeled and fabricated thin film devices, such as changing the perceived colors of thin film stacks^3^ or affecting the plasmonic behavior.^37^ To counter this, there have been efforts to incorporate thickness-dependent effects into the optical design process.^38,39^ Therefore, a proper characterization of fabricated ultrathin films is of utmost importance to determine their actual *ñ* (i.e., both *n* and *k*), and to improve the design and modeling process.

A single intensity measurement, e.g., of either transmittance (*T*) or reflectance (*R*), does not yield sufficient information to determine *n* and *k*. A combination of *T* and *R* measurements can be used, although this requires either a complicated optical setup or multiple measurements in succession. One solution to this problem is the use of either a transmission or reflection measurement with additional information acquired from the phase of light.

Such an approach is followed by ellipsometry,^40,41^ which is a common technique for characterizing the properties of thin films in reflection. The optical response using several polarization states, incident angles, and wavelengths is used, together with a multilayer interferometric model, to estimate the thickness or *ñ*. Classical (standard) ellipsometers use a single measuring spot, with the captured signals being averaged over an area of a few mm^2^. Therefore, only uniform, unstructured layers can be characterized, without capturing lateral spatial information. More advanced imaging ellipsometers^42−45^ can provide spatial information, but they are limited in field-of-view and magnification because of the angled optical setup introducing distortions. Additionally, they tend to be complex and expensive. Therefore, there is an unmet need to characterize the spatial optical response of transparent or semitransparent thin film structures using simple, cost-effective, and integrable techniques.

Digital holography^46−52^ is a quantitative imaging technique that can be used to obtain both the intensity and phase of the complex transmitted or reflected electric field. This information has been used to study sample topography,^53−56^ structures inside a sample (e.g., laser-induced refractive index changes^57,58^), and engineered optical elements (e.g., diffraction and wavefront-shaping meta-surfaces^56^). More specifically, digital holography has also shown potential for the characterization of thin films, e.g., for the dynamics of liquid films,^59,60^ thin film deposition,^56,61^ and chemical etching processes.^54^ Furthermore, a walk-off interferometer^12−14^ has been demonstrated to characterize the phase of light transmitted through metallic films using a set of laser diodes, with the purpose of modeling *ñ* without capturing spatial information, and a quadri-wave lateral-shearing interferometer (QWLSI) has been used in quantitative phase imaging of two-dimensional (2D) materials.^62^ Digital holographic imaging commonly uses an interferometric setup with separated reference and sample beam paths, e.g., using phase shifting^63,64^ or an off-axis setup,^65−67^ requiring a coherent light source, typically a laser. These setups are known to be sensitive to external perturbations, such as vibrations or air turbulences, and their high levels of coherence also create speckle noise or interference artifacts from reflections and scattering on any of the optical components. Since the optical response of thin films depends strongly on the optical wavelength, they need to be characterized by using a broad range of wavelengths, which is difficult to achieve by using the required highly coherent lasers. Large wavelength ranges are easily accessible using a set of light-emitting diodes (LEDs), or by using broadband sources (e.g., halogen, xenon; supercontinuum lasers) together with tunable filters, but these might not offer the required temporal or spatial coherence. This problem is solved by common-path holographic techniques, e.g., phase contrast^68,69^ or differential interference contrast (DIC),^70−75^ both of which are compatible with low coherence illumination sources. They are thus less prone to speckle noise or other interferometric artifacts. Additionally, the common-path setup is inherently stable, allowing for sensitive measurements without the need for expensive vibration dampening.

In previous work, we demonstrated the use of birefringent Savart plates (SP) in a lens-free holographic microscope^58,76−78^ for the differential phase imaging of highly transparent samples. In this work, we demonstrate this lateral-shearing interferometric microscopy (LIM) technique for multispectral intensity and phase imaging of semitransparent films. This specific implementation follows a novel approach, integrating SP into a standard microscope built using off-the-shelf components, enabling the capture of quantitative phase images with nanometer phase sensitivity and micrometer lateral resolution over a large spectral range. Based on this technique, we present a novel optical characterization method for ultrathin semitransparent and absorbing materials. More specifically, we imaged optical responses of samples in the wavelength range from 475 to 750 nm using a supercontinuum white-light laser with a tunable filter with a 10 nm bandwidth. Phase-shifting was used to record a set of interferograms and extract the phase and intensity for the varying wavelengths, and the shearing interferometer transfer function was modeled to reconstruct the absolute phase from the differential phase via deconvolution. Both the measured intensity and phase were verified experimentally by means of a commercial spectrometer and a commercial phase imaging target, respectively.

As a prototypical application and to demonstrate this method for the analysis of semitransparent thin films, we fabricated and characterized cupric oxide (CuO) seeded gold UTMFs^24−26^ with mass-equivalent thicknesses from 2 to 27 nm. These films have peak *T* in the range of 40–80%, depending on the thickness of the gold, and additionally a strong interaction with the phase of the transmitted light. They also display a highly wavelength-dependent response. Therefore, they were ideal for demonstrating the multispectral imaging performance of the proposed LIM technique, both in intensity and phase. By using multilayer interference and a parametric model for the *ñ* of the UTMFs, the measured optical intensity and phase data were then used to determine the *ñ* of UTMFs of varying thickness.

To the best of our knowledge, this is the first work to use multispectral intensity and phase imaging in transmission mode to capture the spatial optical response of ultrathin gold films and to determine their complex refractive index *ñ*. Furthermore, the intensity and phase images could also be used to characterize a sample’s topography, refraction, and diffraction effects. Therefore, we believe the proposed LIM technology could become a relevant tool for the characterization and quality control of optical elements, such as for the design and verification process of multilayer optical coatings, transparent conductors, and meta-surfaces and meta-lenses.

## Multispectral Intensity and Phase Imaging Technique

### Lateral-Shearing Interferometric Microscopy (LIM)

**Figure 1:**
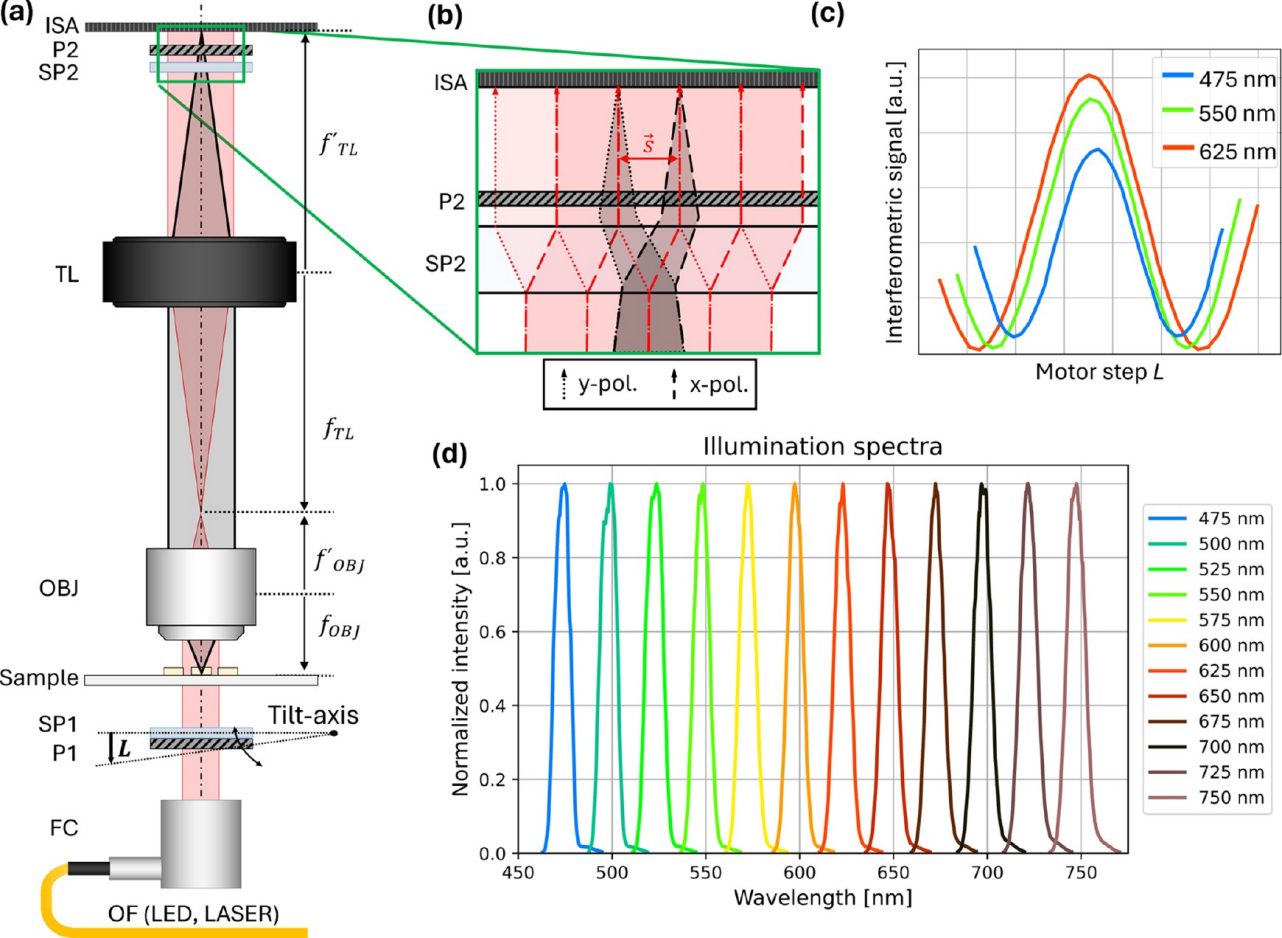
(a) Lateral-shearing interferometric microscopy (LIM) setup consisting of objective (OBJ), tube lens (TL), parallel linear polarizers (P1, P2), Savart plates (SP1, SP2), and image sensor array (ISA). An optical fiber (OF) with a fiber collimator (FC) is used for illumination. Linear motor movement *L* to tilt SP1 and introduce controllable phase shift. (b) Image shearing *s⃗* of orthogonal polarizations (y-pol. dotted, x-pol. dashed) using SP2. (c) Interferogram signals according to the phase delay introduced by tilting SP1. (d) Normalized illumination spectra (FWHM 10 nm) for center wavelengths ranging from 475 to 750 nm.

Figure 1a displays the proposed LIM system, based on a microscope with an effective magnification of *M* ≈ 11, with objective (Olympus PLN 10×, focal length *f'* = 18 mm) and tube lens (TL, Thorlabs TTL-200, *f'* = 200 mm) in a bi-telecentric configuration. For illumination, a supercontinuum laser (NKT Photonics SuperK) with a tunable filter (NKT Photonics Varia) is coupled into an optical fiber (OF), and a fiber collimator (FC; Thorlabs RC12FC-P01) creates an on-axis illumination beam with a plane wavefront.

Two pairs of polarizers (P1 and P2) and Savart plates (SP1, SP2; United Crystals, 50 μm shear) implement the holographic interferometric imaging scheme. P1 and SP1 are placed directly after the collimator and used to split the illumination beam into orthogonal polarizations with a tunable phase retardation. The shear of SP1 is not relevant for image formation and is removed in the scheme for the sake of clarity. In the base configuration, where P1 and SP1 are oriented orthogonal to the collimated beam, there is zero phase retardation between the polarizations. Tilting SP1 by means of a linear motor movement (*L*) introduces a controllable phase retardation between the orthogonally polarized beams, enabling phase-shifting interferometry as described in our previous work.^58,77^ SP2 allows for the formation of the interferometric image. As it is shown in Figure 1b, the SP2 splits and laterally displaces (“shears”) the image into two copies of orthogonal polarizations, which are then interfered through P2, creating a differential shearing interferogram on the image sensor array (ISA). The physical shear of |*s⃗*| = 50 μm affects the magnified image on the camera sensor, and therefore the effective shear as seen in the image is given as |*s⃗*|/*M* ≈ 4,5 μm.

The detected interferogram intensity *I*_ISA_(*r⃗*, *Δϕ*, *θ*) on the ISA is given as
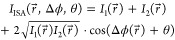
1with *I*_1_ and *I*_2_ being intensities of the beams sheared by SP2, and
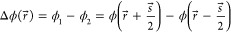
2is the differential phase, where *ϕ*(*r⃗*) is the phase of the beam after transmission through the isotropic, nonbirefringent sample and imaging system and before entering SP2. *s⃗* = (*s*_*x*_, *s*_*y*_) is the shear vector introduced by SP2, and *θ* is the controllable phase shift introduced by tilting SP1, while *r⃗* = (*x*, *y*) denotes the lateral image coordinates of the whole system.

Intensity and phase images are calculated using the phase-shifting interferometry procedure. Herein the phase shift *θ* is varied over a range ≥2π by tilting the SP1 through a linear motor movement *L*, and several interferograms are recorded, typically a number ranging from 20 to 30, from which the intensity image
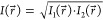
3and differential phase image *Δϕ* are calculated. Finally, *Δϕ* is converted into the corresponding optical path difference (OPD) image
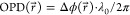
4with *λ_0_* being the (centroid) wavelength of the light used to illuminate the sample.

This procedure is performed both for the sample of interest in the beam path, to get sample *I*_S_ and OPD_S_, as well as for a reference measurement without the sample, to get the reference *I*_R_ and OPD_R_. This allows calculating the sample’s transmittance
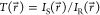
5as well as the OPD image as
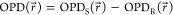
6to compensate for intrinsic phase errors of the system. In the following, we will omit *r⃗* for brevity.

This process is repeated for any wavelength selected using the tunable filter, collecting multispectral data for *T* and OPD. To use this technique with different wavelengths, only the tilt motor range needs to be scaled accordingly, as longer wavelengths require larger movements to achieve the same ≥2π phase-shifting range, which can be seen in Figure 1c where we display the interferogram signals of the illumination beam dependent on motor position for wavelengths of 475, 550, and 625 nm. The normalized illumination spectra used for this work are shown in Figure 1d, ranging from 475 to 750 nm in steps of 25 nm.

**Figure 2:**
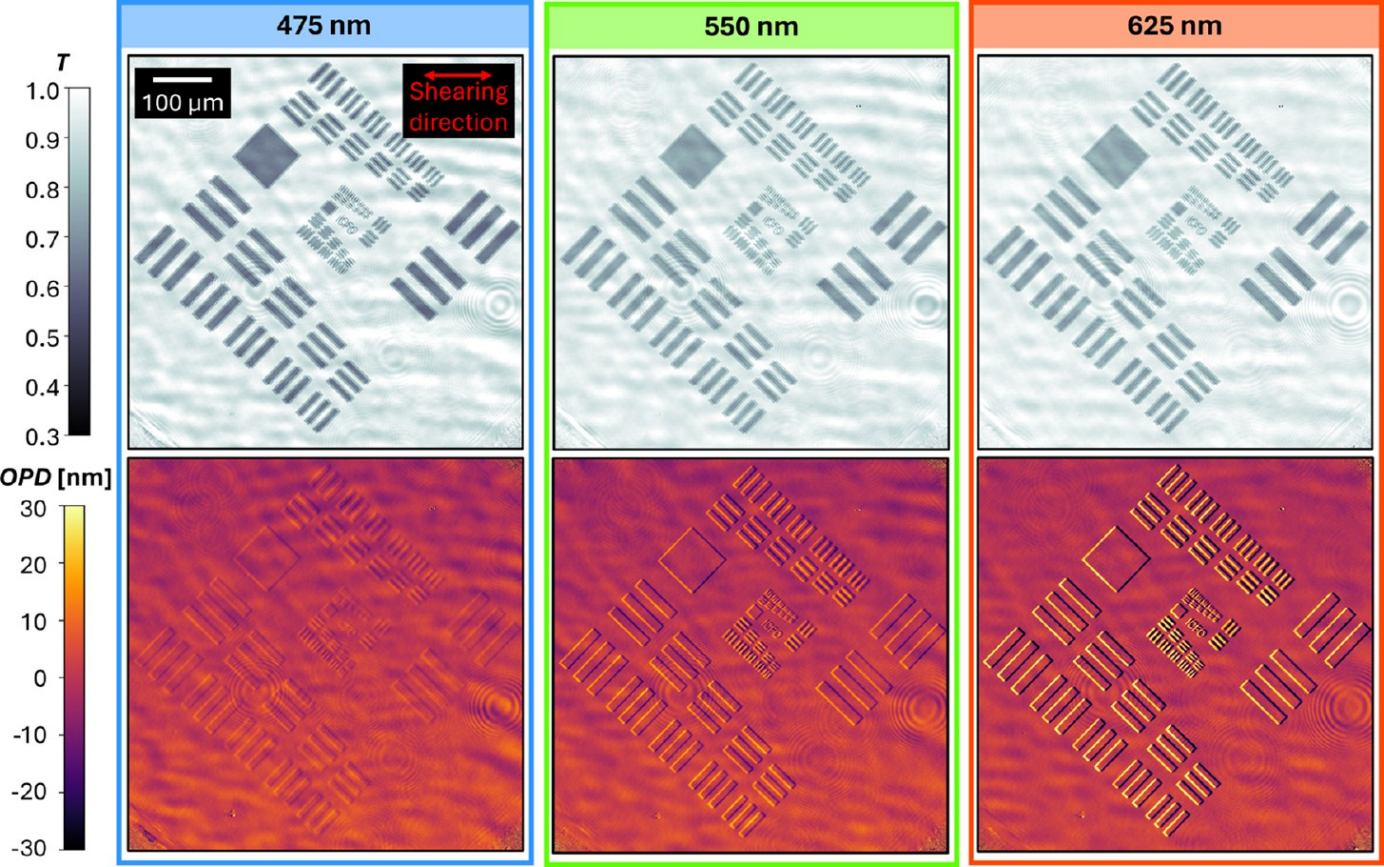
*T* and OPD images of a custom USAF pattern made of a CuO-seeded gold film with *d* = 6 nm, comparing the wavelengths 475 nm (blue), 550 nm (green), and 625 nm (red).

Example *T* and OPD images of a semitransparent USAF pattern made of a gold UTMF (*d* = 6 nm, for details on fabrication and pattern, see the next section of the manuscript, and Section SI-2 of the Supporting Information) are given in Figure 2 for the wavelengths 475, 550, and 625 nm. The semitransparent UTMF structure is visible in both intensity and phase images, demonstrating the ability of the imager to decouple the intensity and differential phase information on the interferograms. It can be observed that both *T* and OPD change with wavelength, demonstrating the need for multispectral imaging. The OPD images show the differential measurement, which for the given examples can be seen as a positive and negative phase “step” at the structures along the shear. For structures of uniform height larger than the shear, the differential measurement gives OPD values of zero in overlapping areas.

### Differential Phase Transfer Function and Phase Reconstruction Algorithm

For applications where the sheared differential effect of the images is detrimental, it is possible to recover a phase image that preserves the original geometry of the sample structures, i.e., without the differential double-image effect. For this procedure, the transfer function of the imager needs to be modeled. The differential phase given by eq 2 can be expressed as a convolution operation with Dirac delta functions δ:
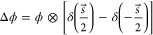
7

In Fourier space, this convolution can be expressed as simple multiplication, relating the Fourier spectra of input phase *ϕ* and differential phase *Δϕ* with the equation
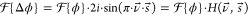
8where *H*(ν⃗, *s⃗*) = 2*i*·sin(π·*ν⃗*·*s⃗*) is the phase transfer function of the shearing operation with Fourier space coordinates *ν⃗ = (ν_x_, ν_y_)*. The shearing transfer function *H*(*ν⃗, s⃗*) has zeros for spatial frequencies fulfilling the relation *ν⃗·s⃗* = *m*, with , i.e., matching spatial frequencies along the shearing direction, including the zero frequency.

Finally, a deconvolution can be performed through means of Tikhonov regularization:^51,79−82^
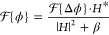
9where H* is the complex conjugate of *H* and  is a regularization parameter. The phase *ϕ* can be calculated from an inverse transform .

This deconvolution operation can be understood as producing an estimate of the original phase in a least-squares sense. Without the regularization parameter, any noise is amplified strongly during the deconvolution. This is especially important for the transfer function *H*(ν⃗, *s⃗*), where there would be division by zero if no regularization parameter was added. Large values of *β* enforce smooth reconstructed images with less noise at the cost of magnitude of signal of the reconstructed features. Small values of *β* give the best representation of the original features of interest; however, the added noise can be detrimental to the image quality. It is common practice^79−81^ to choose *β* empirically while trying to achieve the best trade-off between noise reduction and preservation of the reconstructed image details, for example by defining *β*= 1/SNR using the reciprocal of the signal-to-noise ratio (SNR).^82^

With this approach, for the case of the LIM technique, the SNR can be estimated based on the maximum signal of the OPD image and the background phase noise of the system. OPD signals can cover the range of −300 to 300 nm before phase jumps occur, considering a measurement wavelength of around 600 nm. The estimated background phase noise of our interferometric scheme can be as low as 0.1 nm, as demonstrated in Supporting Information, Section SI-1, which would give an SNR = 600/0.1 nm = 6000. However, this assumes a sample that optimally uses the dynamic range. Typically, the sample OPD signals are in the 10–100 nm range. This holds true for both the phase benchmark sample and UTMFs. Therefore, a more realistic estimation is to use 10–100 nm for the maximum signal, resulting in SNR = 10/0.1 = 100 to SNR = 100/0.1 = 1000, respectively. If one considered the background of a glass slide (which holds the sample structure) as the noise limit, the SNR value could go even lower. For example, approximating an OPD root-mean-square (RMS) of 1 nm for a typical glass slide, then the SNR for signals of 10 or 100 nm is 10 or 100, respectively. The SNR can be visualized as a means to estimate the order of magnitude of *β*. For example, *β* could be determined in a simple trial and error process, and it is impossible to find a value that satisfies all conditions for the sample and background noise. We ultimately choose to use the regularization parameter *β* = 0.001, based on SNR = 1000, i.e., we perform the reconstruction with relatively weak regularization, emphasizing correct reconstruction of the OPD signal magnitudes, this at the cost of additional noise.

**Figure 3:**
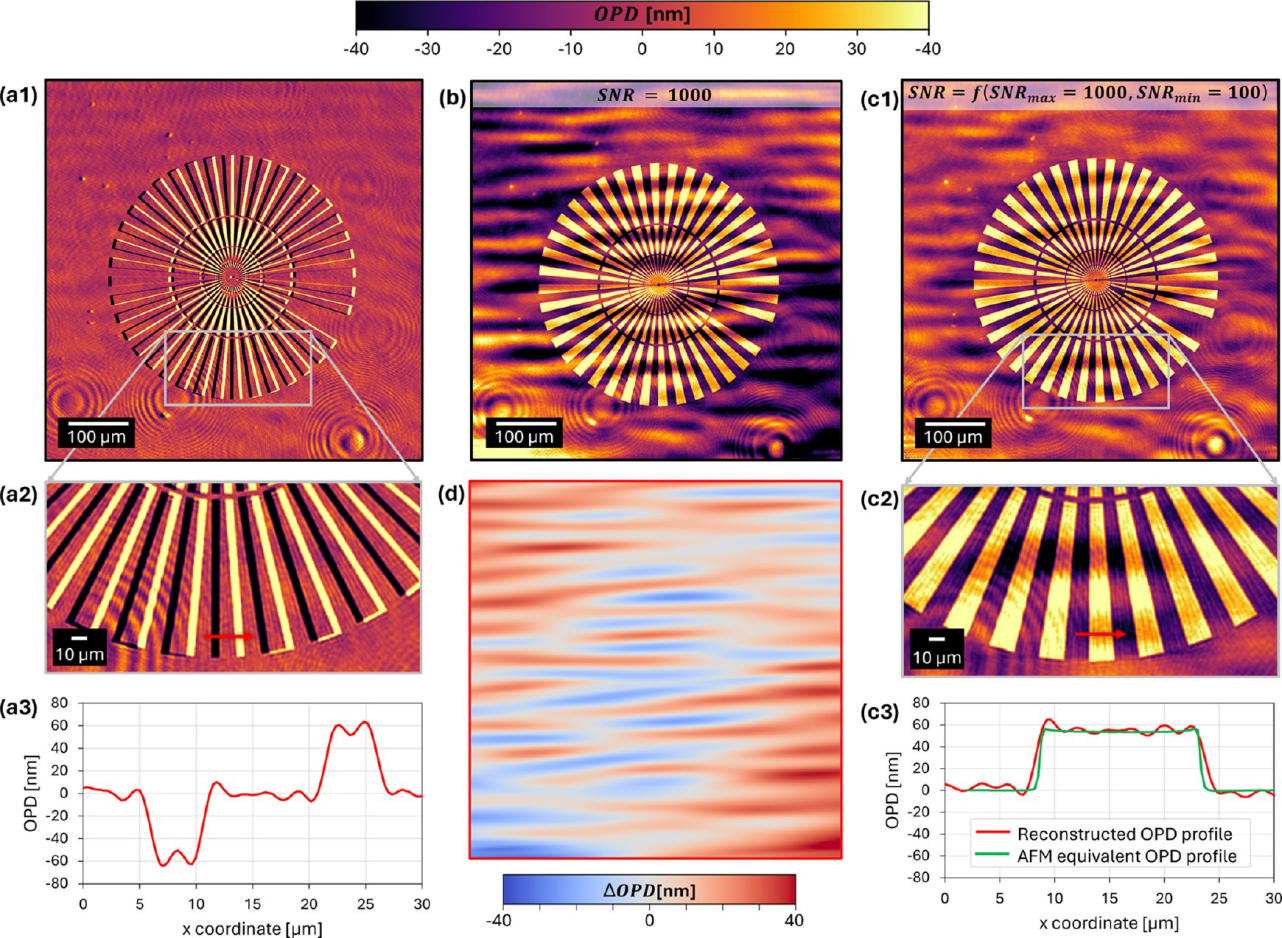
OPD images (*λ_0_* = 675 nm) of a surface relief Siemens star structure (Benchmark Technologies Quantitative Phase Target; polymer, *n* ≈ 1.508 for *λ_0_* = 675 nm; *d* = 109 nm): (a1) Differential OPD image with (a2) zoom-in and (a3) OPD profile. (b) Reconstructed OPD images for constant SNR = 1000. (c1) Reconstruction with custom SNR function with SNR_max_ = 1000, SNR_min_ = 100, *p* = 100, with (c2) zoom-in, (c3) OPD profile (offset corrected) compared to atomic force microscope (AFM) equivalent profile (green). (d) Difference image ΔOPD = OPD_(c1)_ – OPD_(b)_.

To characterize the phase imaging performance of the LIM technique together with the reconstruction algorithm, we measured a transparent surface relief Siemens star (Benchmark Technologies Quantitative Phase Target), with results shown in Figure 3. The nominal height of the structure is 109 nm, and the real part of the refractive index *n* ≈ 1.508 at the measurement wavelength of 675 nm, resulting in an expected signal of OPD = (*n* – *n*_air_)·*d*_s_ = (1.508 – 1)·109 nm = 55 nm for air as the surrounding medium. This estimation is based on a single pass of the light through the structure and is only valid for highly transparent structures. For semitransparent thin films, such as gold UTMFs, a more complex multilayer interferometric model is required to describe the OPD in transmitted light, where multiple reflections and interference effects with changes in intensity and phase are considered.

Figure 3a1 shows the differential OPD image with zoom-in in Figure 3a2, with its OPD profile shown in Figure 3a3, respectively. The profile clearly shows the negative and positive “steps” in the differential measurement scheme, with the profile oscillating slightly around −55 and +55 nm, in agreement with the expected value. In Figure 3b, we show the reconstructed OPD for SNR = 1000, based on the above estimation. The large SNR value, i.e., weak regularization with a smaller *β*, emphasizes correct reconstruction of the image features, however at the cost of more noise, as can be seen by the large background distortions.

To reduce the noise in the reconstruction, without compromising the magnitude of the feature signals, we developed a custom function
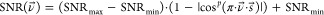
10which gives a frequency-dependent SNR (ν⃗) that uses a lower SNR_min_ in the frequencies around the zeros of the transfer function *H*(ν⃗, *s⃗*), while using a higher SNR for other frequencies, up to a maximum SNR_max_, resulting in better reconstruction results than using an equivalent constant value. Herein, *p* defines the slope and shape of the transition between SNR_max_ and SNR_min_.

The reconstructed OPD image using this custom SNR function with SNR_max_ = 1000, SNR_min_ = 100, and *p* = 100 is shown in Figure 3c1,c2. To show the differences between the two different reconstructions, we calculate the difference image between the OPDs of Figure 3b,c1, displaying the resulting image of ΔOPD in Figure 3d. The custom function generates the same feature OPD signals, which is demonstrated by the fact that the star is not visible in ΔOPD, while at the same time removing the strong background distortions which are in the ±40 nm range.

To verify that the custom function generates the correct OPD signal for the Siemens star, we take an atomic force microscope (AFM) measurement across the bottom bar of the siemens star and calculate the corresponding OPD profile using the known *n*. The AFM profile and the reconstructed OPD are shown in Figure 3c3. Both are in good agreement, demonstrating that the proposed scheme can accurately measure OPD and phase. Verified by this benchmark measurement and for consistency, we chose to use the same custom SNR function for all of the other OPD reconstructions in this work.

## Characterization of Gold UTMFS

As a prototypical example for imaging and characterization of a semitransparent thin film using our proposed LIM technique, we fabricated and analyzed a range of gold UTMFs with (nominal, mass-equivalent) thicknesses ranging from 2 to 27 nm. They have peak *T* values in the range between 40 and 80%, depending on the thickness of the gold, and show a strong interaction with the phase of the transmitted light. In addition, they show a strong dispersion, i.e., the response is strongly wavelength-dependent. Therefore, they are ideal to demonstrate the multispectral imaging performance of the LIM technique, both in intensity and phase. The characterization includes imaging of *T* and OPD, setting up an optical model to relate these to the properties of the UTMFs, and combining the experimental data and the model to determine *ñ* of the films of varying thickness.

The gold UTMFs were fabricated by using a 0.5 nm CuO seed layer, using the same recipe as our previous work^25^ (for more details about deposition, patterning, and quality control, see Supporting Information, Section SI-2). We demonstrated that the use of a 0.5 nm CuO seed layer promotes early percolation and the continuity of the gold UTMFs. The CuO-seeded gold UTMFs start percolating at 1.2 nm thickness, and porosity decreases with thickness, becoming continuous in the range 3–4 nm. The corroborating morphology measurements (AFM and SEM) were also reported in our previous work.^25^ We note that films with a porosity on the scale much lower than the wavelength, in our case, behave like continuous films. For this work, we created a pattern of seeded gold ribbons of 30 μm width, with a spacing of 100 μm, to be able to use the LIM technique to measure the differential phase at the edges of the ribbons. Additionally, the samples include a 15 × 15 mm^2^ large area with a continuous UTMF to allow for measurements using a spectrophotometer and an ellipsometer and to measure sheet resistances. A description of this 2D pattern can be found in the Supporting Information (Section SI-2). Additionally, in Section SI-2, we show sheet resistance and AFM measurements to verify the quality of the UTMFs with regard to our previous work.^25^

### UTMF Intensity and Phase Images

**Figure 4:**
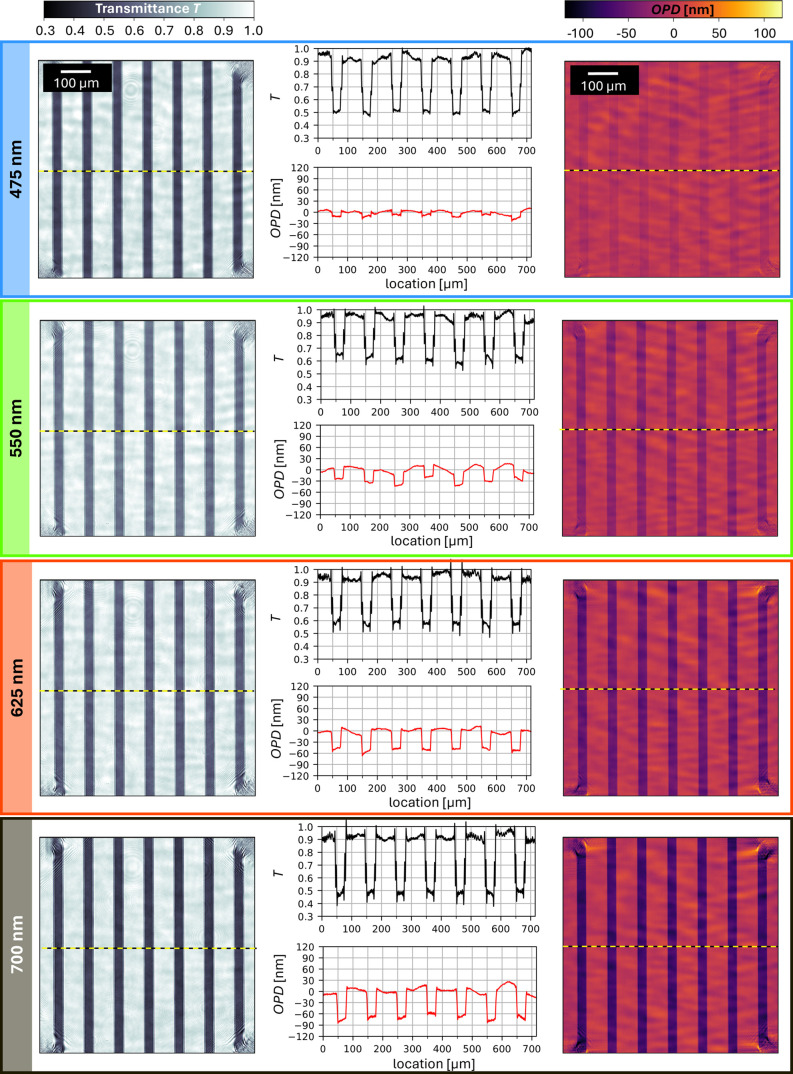
Images for *T* and OPD of seeded UTMF with 12 nm mass-equivalent gold thickness, for wavelengths 475, 550, 625, and 700 nm. The areas covered by the yellow-black dashed lines are used to calculate the profiles shown next to each respective image.

We measured the seeded gold UTMFs with nominal (mass-equivalent) thicknesses of 2, 3, 4, 6, 9, 12, 18, and 27 nm. Illumination wavelengths ranged from 475 to 750 nm, in intervals of 25 nm and with spectral widths set to 10 nm. In order to compensate for the imaging system’s focal shifts due to chromatic aberration, measurements were performed in groups of 3 wavelengths, e.g., 475, 500, and 525 nm for the first group and 700, 725, and 750 nm for the last group, and the focus was adjusted manually for each group. Figure 4 shows a comparison of four exemplary wavelengths out of the whole set with respective *T* and reconstructed OPD images with horizontal profiles visualized below.

### Model of Optical Response and Complex Refractive Index

The measured *T* and OPD images in Figure 4 show how light interacts with the sample. For the characterization of UTMFs it is necessary to have a model that allows one to retrieve information from the measured *T* and OPD. For thin films, this interaction can be simulated using a multilayer interference model, which strongly depends on the structure and the properties of the constituting layers. The model calculates the complex Fresnel coefficients *t̃* and *r̃* for transmitted and reflected light, respectively. By fitting the model parameters to the experimental data sets, we can determine the “best-fit” curves^83^ for *T* and OPD and the complex refractive indices *ñ*_UTMF_ of the gold UTMFs of varying thicknesses.

**Figure 5:**
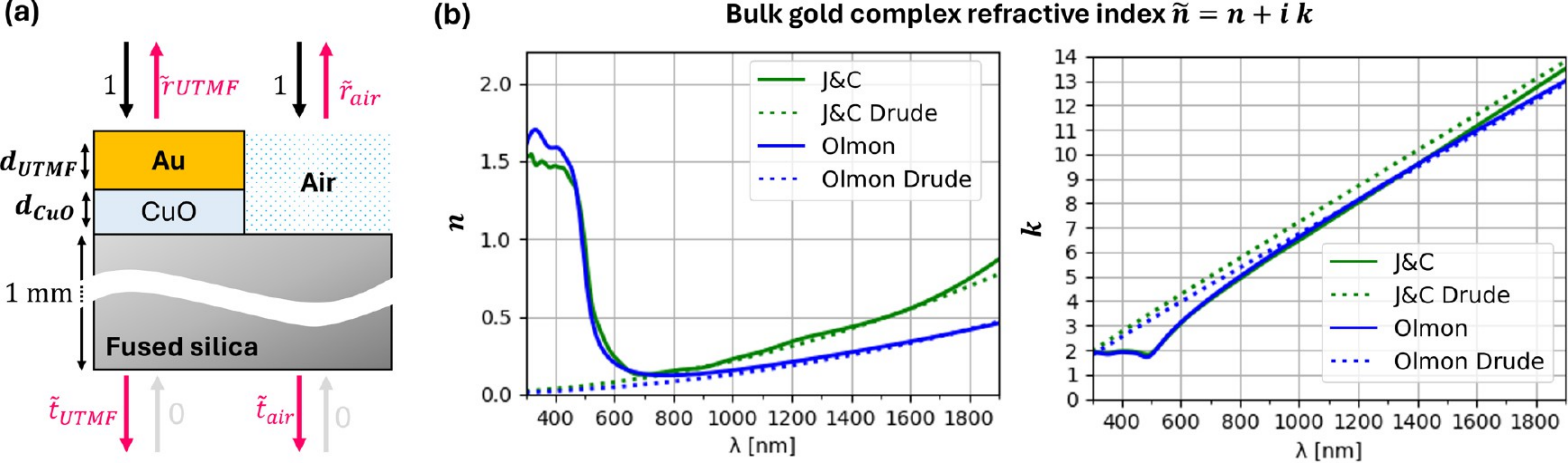
(a) Multilayer model to simulate the complex Fresnel coefficients *t̃* and *r̃*, to calculate *T*_SIM_ and OPD_SIM_ of the UTMF structure. (b) Real part *n* and imaginary part *k* of bulk gold from of Johnson and Christy (J&C),^35^ and Olmon,^34^ with their respective fitted Drude oscillator contributions (dashed), using parameters *ω*_p_ = 9.06 eV/ℏ and *τ*_0_ = 9 fs for J&C^35^ and *ω*_p_ = 8.45 eV/ℏ and *τ*_0_ = 14 fs for Olmon.^34^

To model *T* and OPD as measured by the LIM technique, we calculated the complex Fresnel coefficients of the seeded UTMFs on the fused silica substrate (*t̃*_UTMF_, *r̃*_UTMF_), using the multilayer model shown in Figure 5a with the transfer matrix method (TMM).^27^ The simulated *T* of light transmitted through the multilayer stack of UTMF and substrate can be calculated as
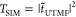
11

Since the phase is measured differentially at the edge of the ribbons, we also simulated the complex Fresnel coefficient for a layer of air (*t̃*_air_, *r̃*_air_) with the same thickness as the UTMF on the substrate. We then calculated the optical path difference
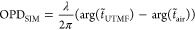
12at the edge of the UTMF. Using this approach, both *T*_SIM_ and OPD_SIM_ are then representative of the *T* and OPD values as measured with the LIM technique.

This model requires *ñ* and thicknesses of all layers, including the surrounding medium and the substrate. Refractive indices of most dielectric materials are commonly available as tabulated data or as parametric models. In our case, the indices of the fused silica substrate are well described by Sellmeier equations.^84^ As demonstrated in our previous work,^25^ the 0.5 nm CuO seed layer promotes early percolation of the gold UTMFs, but its contribution is negligible to the optical transmission and reflection in the visible and infrared range. In Section SI-6 of the Supporting Information, we confirm this numerically. We therefore omit it from the simulation model (*d*_CuO_ = 0 nm).

The nominal UTMF thickness, *d*_UTMF_ is given by the mass-equivalent thickness of the deposition process. For thick gold films, tabulated data of *ñ* is available in the literature.^34,35^ However, the thinner the film, the higher the dependence of *ñ* on thickness. This is especially the case for UTMFs with thicknesses of ≤10 nm. We define a parametric model for the complex dielectric constant *ε̃*_r,UTMF_(*ω*) and the resulting  of the gold UTMF. As it was discussed in the literature,^21,34,35,85−87^ the *ε̃*_r_(*ω*) of metals is determined by the interaction of light with the bound and free electrons:
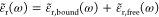
13

The free electron interactions can be described by a Drude oscillator model:
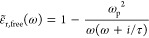
14with plasma frequency *ω*_p_ and scattering time *τ*. The Drude oscillator model properly describes *ñ* in the infrared range. This is corroborated by Figure 5b, comparing *n* and *k* of bulk gold as tabulated by Johnson and Christy^35^ and Olmon^34^ with their corresponding Drude components. To be able to describe the *ε̃*_r_(*ω*) over the whole visible spectral range, one also needs to take the bound electron contribution *ε̃*_r,bound_(*ω*) into account. As shown in the literature,^24,25,87^ it is possible to model the bound electron contributions using the tabulated bulk data *ε̃*_r,bulk_(*ω*) and subtracting its Drude component with the known parameters *ω*_p,bulk_, *τ*_bulk_:
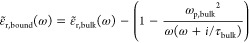
15

To describe the final parametric *ε̃*_r,UTMF_(*ω*, *ω*_p,UTMF_, *τ*_UTMF_) of the UTMF, we then insert eqs 14 and 15 into eq 13, and get
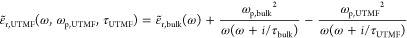
16from which we calculate 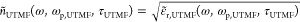
To adapt this model to UTMF of varying thickness, *ω*_p,UTMF_ and *τ*_UTMF_ are taken as variable parameters, whereas the same tabulated *ε̃*_r,bulk_(*ω*) and bulk parameters (*ω*_p,bulk_ = 8.45 eV/ℏ and *τ*_bulk_ = 14 fs) by Olmon^34^ are used to describe the *ε̃*_r,bound_(*ω*), independent of the UTMF thickness.

### Determination of Complex Refractive Index from Experimental *T*, OPD

**Figure 6:**
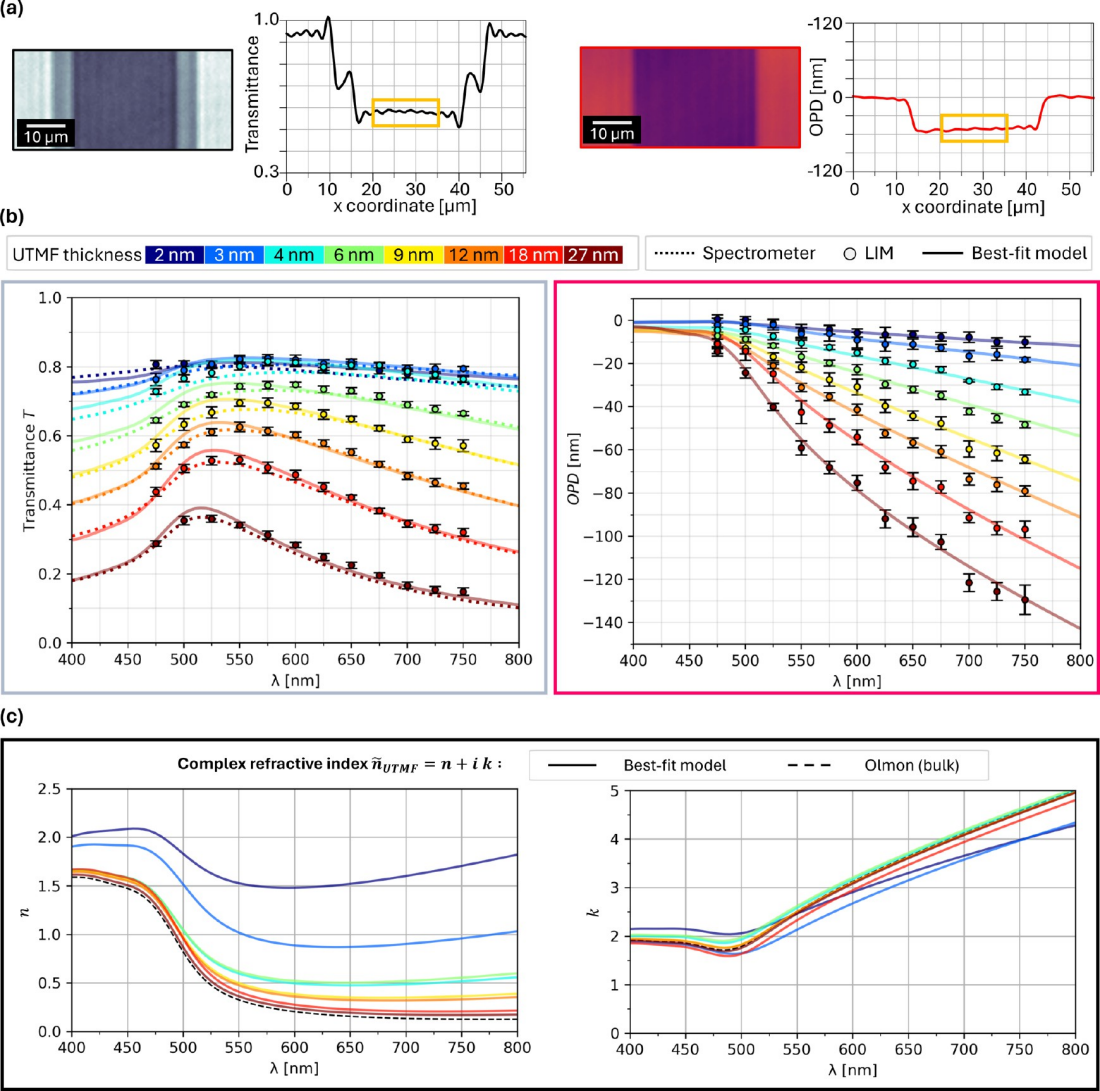
(a) Scheme for the analysis of *T* and OPD: Enlarged views with averaged profiles along *y*-axis. Central data points (yellow box) are averaged to assign the *T* and OPD values. (b) Multispectral *T* and OPD of UTMFs of varying thicknesses: Comparison of experimental data sets (*T*_LIM_, OPD_LIM_; circles; error bars ±1*σ*_LIM_ standard deviation), spectrometer (dotted) and best-fit optical model (*T*_SIM_, OPD_SIM_; solid). (c) Determined *ñ*_UTMF_ = *n* + *i k* for the investigated UTMF thicknesses.

In order to create experimental data sets *T*_LIM_ and OPD_LIM_ to fit the optical model, average and standard deviations are calculated using values collected from 10 areas on the UTMF ribbons. Exemplary enlarged views of *T* and OPD for this procedure are shown in Figure 6a, from which a profile is calculated by averaging along the *y*-axis. For the OPD profile, we additionally remove any background tilt and offset. From these profiles, the final values for *T*_LIM_ and OPD_LIM_ are calculated by averaging the central data points marked by the yellow box. This analysis was performed for all measured UTMF thicknesses, with the previously described illumination wavelength range from 475 to 750 nm.

The resulting average *T*_LIM_ and OPD_LIM_ values are displayed in Figure 6b, with error bars indicating the 1*σ* standard deviation. For this set of UTMF thicknesses, it can be observed that *T*_LIM_ decreases with increasing thickness, additionally changing from a flat curve to showing a more pronounced peak between 500 and 600 nm. To validate the accuracy of the *T*_LIM_ values in Figure 6b, we measured the samples with a commercial spectrometer (dotted lines; PerkinElmer LAMBDA 950), and as can be seen, both data sets are in good agreement. The OPD_LIM_ data show a consistent trend in the observed wavelength and UTMF thickness range. OPD_LIM_ values are quite flat below 500 nm, with the magnitude of the OPD_LIM_ then scaling approximately linearly with increasing wavelength and at the same time increasing with the UTMF thickness.

The solid lines in Figure 6b are the best-fit *T* and OPD curves which are determined by fitting the optical model parameters (plasma frequency *ω*_p,UTMF_, scattering time *τ*_UTMF_) to the experimental data by minimizing the error^83^
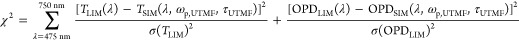
17between experimental data set (*T*_LIM_, OPD_LIM_) and simulated data set (*T*_SIM_, OPD_SIM_), weighted with the respective standard deviations σ(*T*_LIM_) and σ(OPD_LIM_).

The resulting best-fit parameters for the measured thicknesses of UTMF are displayed in Table 1 together with the respective standard deviations *σ* that were given by the fitting algorithm.^83^ The fitted plasma frequencies vary around the expected bulk plasma frequency *ω*_p,bulk_ = 8.45 eV/ℏ. The scattering time *τ*_UTMF_ displays a linear increase with the thickness. In Section SI-3 of the Supporting Information, we compare these values to parameters published in recent studies and show that *τ*_UTMF_ is expected to slowly approach the bulk scattering time *τ*_bulk_ = 14 fs for thicknesses larger than those investigated here.

**Table 1:** Best-Fit Thickness-Dependent Parameters for Drude Component of Gold UTMF

*d* [nm]	ℏ*ω*_p,UTMF_ [eV]	*σ*(ℏ*ω*_p,UTMF_) [eV]	*τ*_UTMF_ [fs]	*σ*(*τ*_UTMF_) [fs]
2	8.70	0.08	0.55	0.03
3	7.86	0.06	1.11	0.04
4	8.54	0.06	2.38	0.14
6	8.61	0.09	2.22	0.19
9	8.42	0.05	3.53	0.29
12	8.42	0.05	3.92	0.39
18	8.17	0.03	6.84	1.01
27	8.38	0.03	9.04	2.05

The *ñ*_UTMF_ = *n* + *i k* given by these parameters are visualized in Figure 6c, where it can be observed that especially the real part *n* shows a strong dependence on thickness, with smaller thicknesses having a higher *n*. While a dependence can also be observed for the imaginary part *k*, its changes with thickness are less pronounced. In Section SI-4 of the Supporting Information, we show that these RI agree with values obtained using a commercial ellipsometer. In Section SI-5 of the Supporting Information, we additionally demonstrate that our optical model and the fitted complex refractive indices can be used to simulate the reflectance of the UTMF samples, showing good agreement with a spectrometer measurement. The transmission-mode characterization, as demonstrated in this work, therefore can also be used to infer the reflectance of a thin film. Overall, these results show that the proposed lateral-shearing interferometric microscopy (LIM) system is suitable to characterize (semi-) transparent samples in the visible range, in our specific case study, the UTMFs of varying thickness, and that optical models can be used to determine sample properties, such as thicknesses or dielectric functions, from the measured intensity and phase information.

## Discussion

### Limitations of the LIM Technique

LIM inherently measures the phase differentially, with the shearing transfer function defined in eq 8. As a result of this, the system suppresses very low spatial frequencies, phase information cannot be measured for uniform films, and information is lost for spatial frequencies that match the roots of the transfer function. The interplay between shear direction, magnitude, and sample structure needs to be considered when applying the LIM technique and interpreting the results. Additionally, since the technique is based on orthogonal polarizations, birefringence in the sample affects the measurements. The measurement principle itself will work, but this needs to be considered when interpreting results.

When applying the phase reconstruction algorithm, one needs to consider that this gives the optimal estimate of an ill-posed inversion problem; i.e., there are spatial frequencies that are suppressed and cannot be recovered. At the same time, errors are introduced. The quality of the reconstruction can significantly vary with the regularization parameter, which might have to be optimized between different samples in order to achieve good results. Additionally, the phase reconstruction will give wrong results for birefringent samples, as the underlying transfer functions are assuming isotropy.

One also needs to keep in mind some limitations when acquiring multispectral data, as presented in this work. Since the multispectral data are extracted from images, one needs to guarantee the same image quality over the whole spectral range. This is historically a challenge in imaging systems and also applies to our technique. More specifically, the employed lenses in our setup were achromatic; i.e., we had to correct for focus shifts when switching illuminations over the whole spectral range. This problem could be alleviated by using apochromatic or superapochromatic lenses.

### LIM vs Ellipsometry for the Determination of Complex RI

Neither our presented method nor ellipsometry are direct “measuring tools” for complex RI. Ellipsometry measures the relative phase change *Δ* and amplitude ratio *Ψ* between the orthogonal polarization components of the beam reflected by a sample. However, *Δ* and *Ψ* on their own provide little information. One must define a multilayer interferometric model based on Fresnel reflection and transmission coefficients must be defined, after which the model parameters (thickness and complex RI) can be fitted to the measured *Δ* and *Ψ*. The current state-of-the-art technology is “spectroscopic ellipsometry” (SE), which measures *Δ* and *Ψ* over a large wavelength range from the ultraviolet (UV) to the near-infrared (NIR), allowing for more stability in the model regression and for several material properties to be determined.^40^

Therefore, the overall complexity of both LIM and ellipsometry and associated modeling and fitting procedures can be considered comparable. For their initial measurements, both require optical elements to control and modulate the polarization states. In addition, both require signal processing for the reconstruction of intensity and phase information, as well as modeling and fitting to determine material properties.

However, there are some key differences between both techniques. In SE, the whole spectral *Δ, Ψ* can be acquired in seconds,^40^ as the point-wise measurement allows the use of a spectrometer in the detection mechanism. However, as mentioned previously, *Δ* and *Ψ* on their own are not very useful, and the data acquisition is limited to a single point of a sample. Inside this measuring spot, a uniform film is required. Furthermore, one-dimensional (1D) profiles or 2D data can be acquired only by scanning the sample.

The proposed LIM technique determines transmittance and phase, which are more directly related to the properties of the material, and the imaging capabilities capture 2D information without the need to scan the sample. The LIM technique inherently measures phase differentially; e.g., phase information is lost for uniform films or spatial frequencies that match the roots of the transfer function. Additionally, spectral information needs to be acquired sequentially, as presented in this work. Lastly, the images need to be analyzed to be able to extract correct *T* and OPD data sets, where the size and shape of the investigated structures can also have an influence.

To summarize, depending on which type of structure and film is to be investigated, one technique or the other may be more appropriate. For the sole purpose of determining the refractive index of a uniform film, the SE technique offers faster data acquisition over a larger spectral range and with a higher spectral resolution. If, instead, 2D information is required in addition to the RI, then the LIM technique becomes more advantageous.

### Limitations in Modeling Fresnel Coefficients and Fitting Refractive Indices

In order to determine the complex RI using the proposed methods, the first step is to define a model of the multilayer structure. The accuracy of the calculated Fresnel coefficients and resulting fitting procedures depends on the model. It requires knowledge of *ñ* and thicknesses of all layers, including the surrounding medium and the substrate. Therefore, this approach can be applied only if the sample structure is already known a priori, at least to an adequate level. Generally speaking, the accuracy of the Fresnel calculations scales with the complexity of the model. Basic models assume perfect layers with well-defined interfaces, while additional roughness layers and transition layers might be used to improve the accuracy of the models. Refractive indices of thin films can vary between different fabrication methods and thicknesses (as shown in this work). For real-world samples, where some but not all parameters of the multilayer model are known, one may consider an iterative process, starting with a basic model with limited information and iteratively increasing the model complexity.

When fitting the refractive indices using this approach, the accuracy will also depend on the underlying dielectric model that is used. For example, in our case, the model depends on bulk refractive index data, with many available in the literature.^34,35^ Alternatively, one could describe the RI based on a fully analytical model, which generally tries to replicate the bulk values.^21,85−88^ This problem is also shared with ellipsometry, where it is a challenge to define a correct dielectric model based on oscillators, which is why mathematical B-splines are often used.

Lastly, the model fitting generally provides a result based on local minimization. Therefore, the final solution may be only a local minimum, and the outcome can depend on the initial starting parameters. This is a general challenge for optimization problems and also applies to ellipsometry.

Nevertheless, despite these various limitations, this modeling approach can achieve results that are in very good agreement with measured experimental data sets, within experimental errors.

## Summary

In this work, we presented a novel method for the characterization and imaging of the optical response of semitransparent thin films. The method uses a lateral-shearing interferometric microscopy (LIM) technique to measure the intensity and phase images accurately and simultaneously. As a prototypical application, we measured the optical properties of ultrathin metal films (UTMFs) of varying thickness and determined their complex refractive indices (*ñ* = *n* + *i k*).

The presented LIM technique uses phase-shifting interferometry to extract both transmittance (*T*) and optical path difference (OPD) images over the visible and near-infrared spectral ranges. Using deconvolution, the original phase is reconstructed from the differential phase, verifying the accuracy of the reconstructed phase profiles with AFM measurements. We fabricated structured cupric oxide (CuO) seeded gold films in several (nominal, mass-equivalent) thicknesses between 2 and 27 nm on fused silica substrates and characterized their optical response in the visible spectral range from 475 to 750 nm, using a supercontinuum laser together with a tunable spectral filter. The measured *T* was shown to be in good agreement with measurements using a commercial spectrometer. We set up a simulation model for the optical response of the UTMF based on multilayer thin film interference using a parametric model of *ñ*_UTMF_(*ω, ω*_p,UTMF_, *τ*_UTMF_). Lastly, the model parameters (*ω*_p,UTMF_, τ_UTMF_) were fitted to the measured *T*, OPD data sets to determine *ñ*_UTMF_ of the UTMFs of varying thicknesses, with *ñ*_UTMF_ matching those obtained from ellipsometry.

## Conclusions

To the best of our knowledge, this is the first work to apply multispectral lateral-shearing interferometry in transmission mode to measure the optical response of ultrathin gold films and to determine their *ñ*_UTMF_. Overall, the LIM technique shows high potential for imaging and characterization of the optical properties of (semi)transparent thin films, using the combined intensity and phase information to determine the films’ properties. Spectral ellipsometry, which some would consider state-of-the-art for thin film analysis, has its advantages over the LIM technique with regard to spectral range, spectral resolution, and acquisition speed. However, the LIM technique can be used in transmission mode and capture spatial information on structured samples. Moreover, compared to other holographic imaging techniques, the LIM technology is based on off-the-shelf components and is compatible with a large range of light sources, making it potentially more cost-efficient and allowing for integration in a range of optical setups, either as a stand-alone system as demonstrated in this work, or as an add-on to commercial microscopes. Based on the presented experiment results and discussion, we believe the LIM technique could become a valuable tool in the design and verification process of surfaces with films, such as optical coating multilayer stacks, transparent conductors, 2D materials, meta-surfaces, and meta-lenses.

## Data Availability

All processed or raw data underlying the results presented in this article are stored at ICFO servers and can be provided upon reasonable request to the corresponding authors.

## Supporting Information Available

Background phase noise (*λ_0_* = 675 nm) (SI-1); UTMF fabrication details and quality control (SI-2); comparison of fitted UTMF scattering times τ with the literature (SI-3); comparison of fitted RI with ellipsometer results (SI-4); comparison of reflectance measured by spectrometer and simulation based on fitted parameters (SI-5); and influence of CuO seed layer on simulated *T*, OPD, *R* (SI-6) (PDF)
