# Supplementary material for: Multispectral Holographic Intensity and Phase Imaging of Semitransparent Ultrathin Films
Source: ACS Photonics. 2024 Apr 30;11(5):1873–86. doi: 10.1021/acsphotonics.3c01834 (PMC11100288; doi:10.1021/acsphotonics.3c01834)
Supplement: Supplementary file 1 — ph3c01834_si_001.pdf [file ph3c01834_si_001.pdf]

Multispectral Holographic Intensity and Phase Imaging of Semitransparent Ultrathin Films

Sebastian Haegele[1,*], Daniel Martínez-Cercós[1], Javier Arrés Chillón[1],

Bruno Paulillo[1], Roland A. Terborg[1], Valerio Pruneri[1,2,*]

[1] ICFO-Institut de Ciències Fotòniques, The Barcelona Institute of Science and Technology, 08860 Castelldefels (Barcelona), Spain

[2] ICREA-Institució Catalana de Recerca i Estudis Avançats, 08010, Barcelona, Spain

<u>Corresponding authors</u>: sebastian.haegele@icfo.eu ; valerio.pruneri@icfo.eu

<u>Supporting information:</u>

9 Pages (S1 to S9); 7 Sections (SI-1 to SI-7); 6 Figures (Fig. S1 to S6)

SI-1. BACKGROUND PHASE NOISE ($\lambda_0 = 675$ nm)

To estimate the optical path difference (OPD) noise-floor of the lateral-shearing interferometric microscopy (LIM) technique, we perform two consecutive reference measurements (i.e. empty beam path without a sample), which are shown in Fig. S1 a) and b) over an area of $1900 \times 1900\ \mathrm{px}^2$, equivalent to approximately $590 \times 590\ \mu\mathrm{m}^2$. As described in the manuscript, we generally use a reference measurement to compensate every sample measurement for intrinsic OPD errors and to calculate the transmittance of a sample.

For this noise analysis, the difference between the two reference measurements determines the *OPD* noise-floor, resulting from the noise sources that cannot be removed by the subtraction operation, e.g. image sensor noise or intensity and phase noise of the illumination. Fig. S1 c) shows the raw OPD noise, with a standard deviation over the whole image of $\sigma \approx 0.7$ nm. As can be seen, the image is dominated by high frequency noise. Fig. S1 d) shows the OPD noise processed with a gaussian smoothing filter ($\sigma_{gauss} = 2.5$ px), resulting in a standard deviation over the whole image of $\sigma \approx 0.1$ nm.

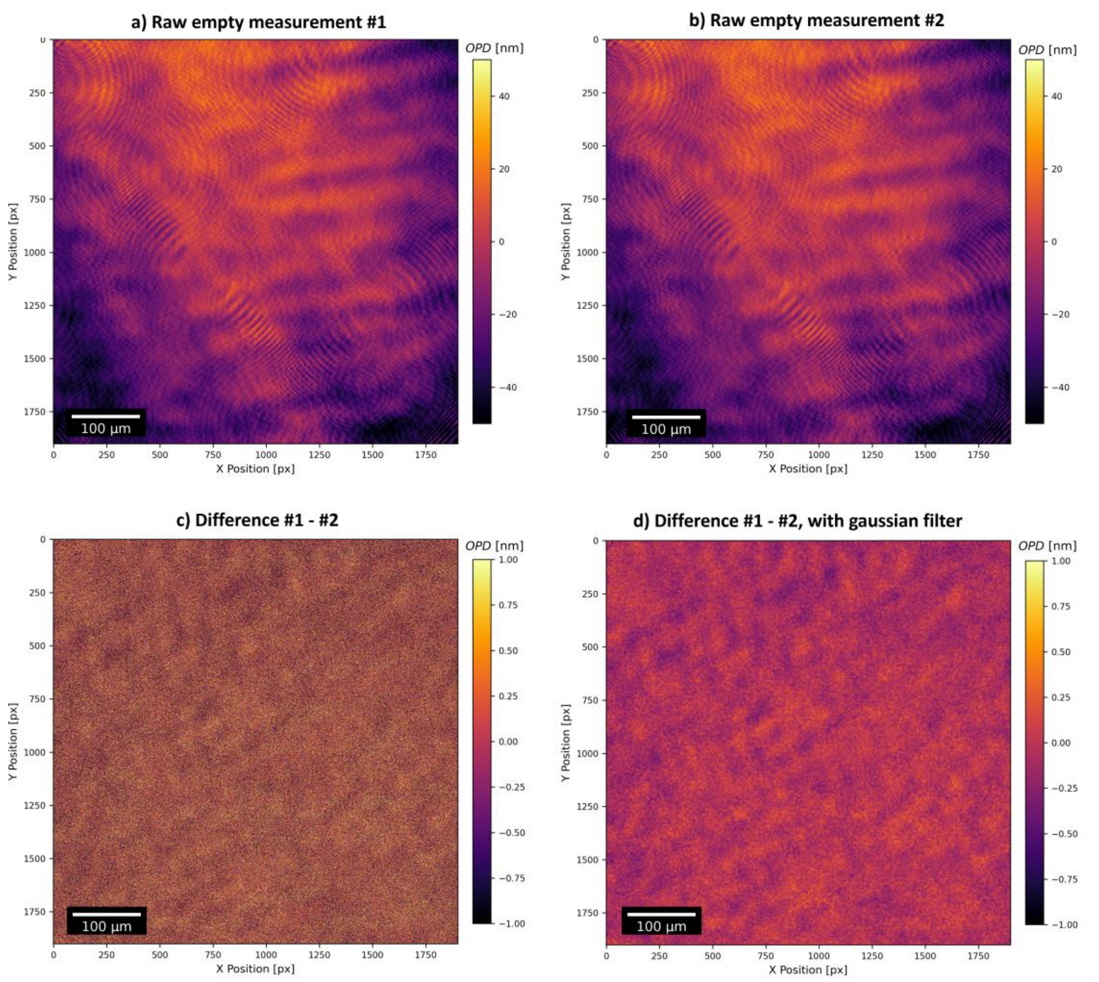

Fig. S1: (a),(b) T*wo consecutive reference measurements (i.e. empty beam path without a sample),* with (c) *OPD* difference and (d) gaussian filtered *OPD* difference, defining the o*ptical path difference (OPD) noise-floor of the lateral-shearing interferometric microscope (LIM)*

SI-2. UTMF FABRICATION DETAILS AND QUALITY CONTROL

A fabrication method described in our previous work[1] was used: the fused silica substrates (high purity fused silica (HPFS); Corning Inc.) are cleaned with acetone in an ultrasonic bath followed by isopropanol rinsing (5 min for each process) and drying with a nitrogen flow. The substrates are first exposed to an O_2 plasma in a Plasma Asher Tepla 300 (bias power 150 W, O_2 flow 200 ml/min) for 3 min. The patterns were carried out using standard UV lithography of an S1805 photoresist on an HPFS substrate previously coated with a hexamethyldisilazane (HMDS) primer. Then the desired patterns are UV exposed with a Heidelberg Maskless Aligner (MLA) 150 followed by development on MF319. The prepatterned photoresist on HPFS substrates are exposed to an O_2 plasma with the same parameters as before and introduced in the sputtering chamber, previously evacuated to a base pressure of about 10^{-7} – 10^{-8} Torr, for the cupric oxide (CuO) deposition. A cupric oxide (CuO, 99.7% purity) sputtering target is used for seed layer deposition with an RF power of 150 W and a working pressure of 2 mTorr in an argon and oxygen atmosphere (20 and 2 sccm, respectively). Oxygen is added to compensate for the possible reduction of the CuO molecules during sputtering. The extrapolated deposition rate is 0.25 Å/s, corresponding to to 20 s deposition time for 0.5 nm of CuO. During the CuO seed deposition, the substrate holder rotates at a speed of 60 rpm. The substrates are immediately transferred to a separate vacuum chamber for gold UTMF deposition via thermal evaporation. Evaporation rate is 1 Å /s, in-situ measured by a quartz crystal microbalance. Morphology measurements (AFM and SEM) for these type of UTMF samples were reported in our previous work[1].

Fig. S2 (a) shows a scheme of the fabricated 2D UTMF patterns. The ribbons and USAF are used for the LIM measurements. The large uniform square is used for several measurements, including sheet resistance, optical transmittance (see transmittance data in manuscript) and ellipsometry (see section SI-4). The sheet resistance measurements, displayed in Fig. S2 (b), are used to compare and verify the quality of the UTMF with respect to previous work[1]. Fig. S2 (c1-3) shows a detailed AFM characterization of bars of the USAF test structure of the 27 nm (nominal thickness) UTMF. Fig. S2 (d1-3) shows a similar AFM measurement on a ribbon structure for 6 nm (nominal thickness). The extracted AFM thickness values for 6 nm, 9 nm, 18 nm and 27 nm UTMF are shown in Fig. S2 (e), showing consistency between mass-equivalent and actual measured thicknesses.

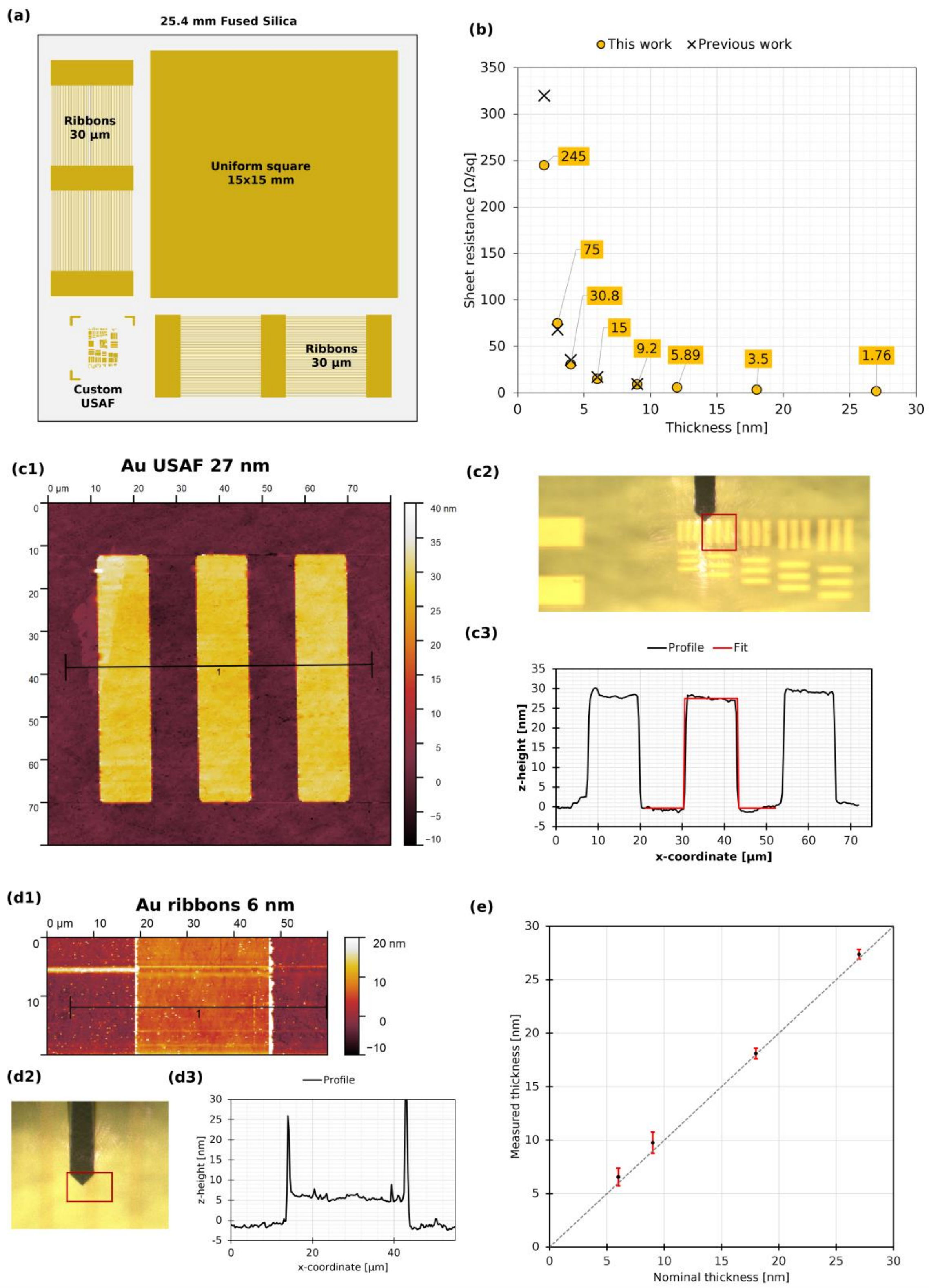

Fig. S2: (a) Scheme of UTMF pattern on 1" HPFS. (b) UTMF sheet resistances compared to previous work [1].(c) AFM measurement of 27 nm Au: (c1) topography of USAF bars, with (c2) corresponding AFM camera image and (c3) extracted profile (black) with fitted shape (red) for height determination. .(d) AFM measurement of 6 nm Au: (d1) topography of ribbon, with (d2) corresponding AFM camera image and (d3) extracted profile. (e) Measured vs nominal mass-equivalent UTMF thicknesses.

SI-3. COMPARISON OF FITTED UTMF SCATTERING TIMES τ WITH LITERATURE

The parameters of the dielectric model of the UTMF films shown in this work (See section 4. of the main document) can be compared with other published values. For example, Mendoza-Herrera et. al. [2] used the same parametric dielectric model and fitted it to tabulated dielectric constants that were measured with ellipsometry by Yakubovsky et al.[3,4] for a range of gold UTMF: 4.1 nm, 6.1 nm, and 9.0 nm films were fabricated on a single-layer MoS_2 seed on silicon wafers with a 285 nm thick SiO_2 top-layer [4], whereas 25 nm, 53 nm and 117 nm films were deposited on a Silicon wafer without any adhesion layer [3], both using electron beam evaporation. Additionally, for this comparison we manually read out the damping constants γ given by Yakubovsky et. al in Fig. 5 of their original publication[3], in order to calculate the corresponding scattering times $\tau = 1/\gamma$.

Figure S2 compares the fitted scattering times τ, where one can see a good agreement between our data and work published by other authors, despite the differences in substrates, seed layers and fabrication technique. The overall trend for the dependent scattering time $\tau(d)$, from thin film to the bulk material, can be estimated with the empirical formula $\tau(d) = [1/\tau_{bulk} + v_F/d]^{-1}$, where $v_F = 1.4 \cdot 10^6$ m/s is the fermi velocity for gold, $\tau_{bulk} = 14$ fs is the bulk scattering time of gold given by Olmon[5] and d is the UTMF thickness.

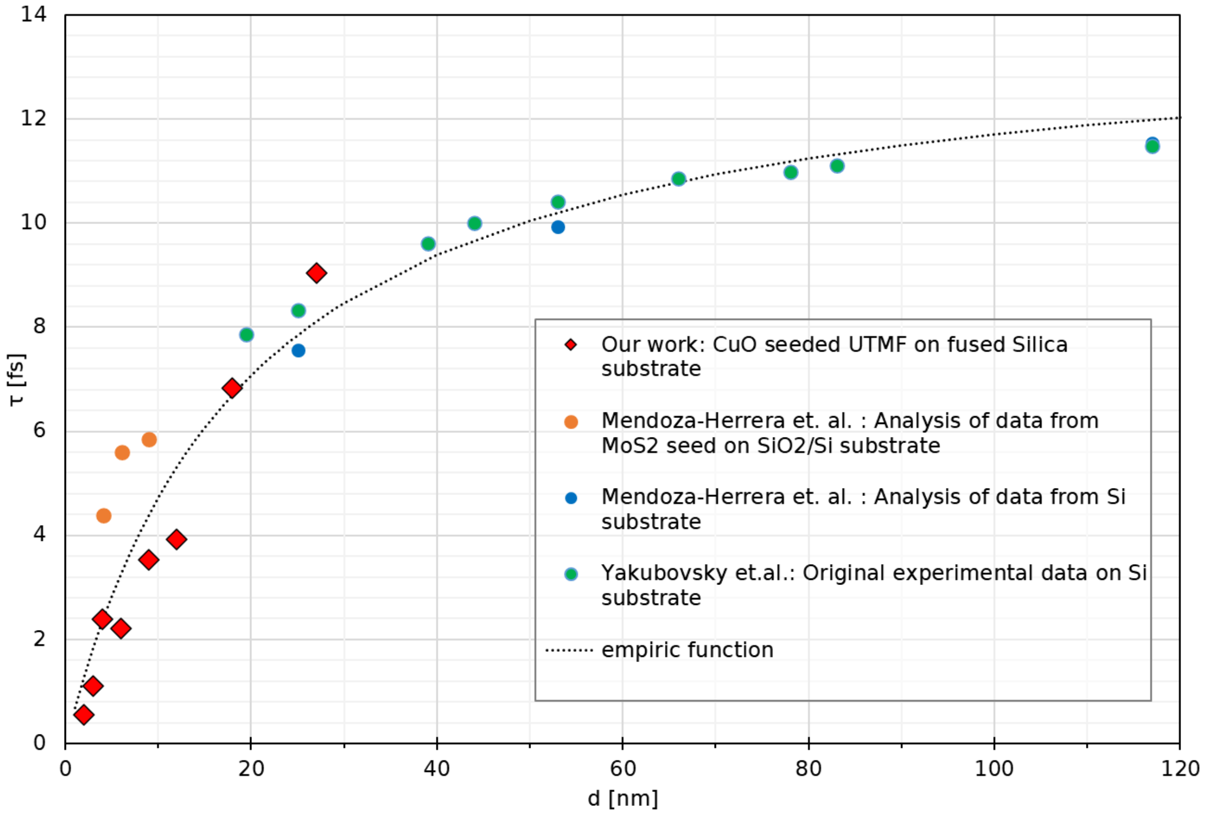

Fig. S3: Comparison of our fitted scattering times τ to published values by Mendoza-Herrera et. al. [2] *and Yakubovsky et al.*[3,4]

SI-4. COMPARISON OF FITTED RI WITH ELLIPSOMETER RESULTS

Fig. S3 shows a comparison of the complex RI values obtained with the presented LIM method and values obtained with a commercial “variable angle spectroscopic ellipsometer” (J.A. WOOLLAM RC2 VASE). Ellipsometric measurements were performed on the 15x15 mm² gold film area shown in Fig. S2, using a spectral range from 300 nm to 1500 nm and using incidence angles of 55°, 65° and 75°. We used the same simple model with the nominal thickness Au on fused Silica, without a CuO seed-layer. The complex RI was fitted using a Kramers-Kronig consistent B-spline method. As can be seen, both techniques give comparable results, with better agreement for thicker films.

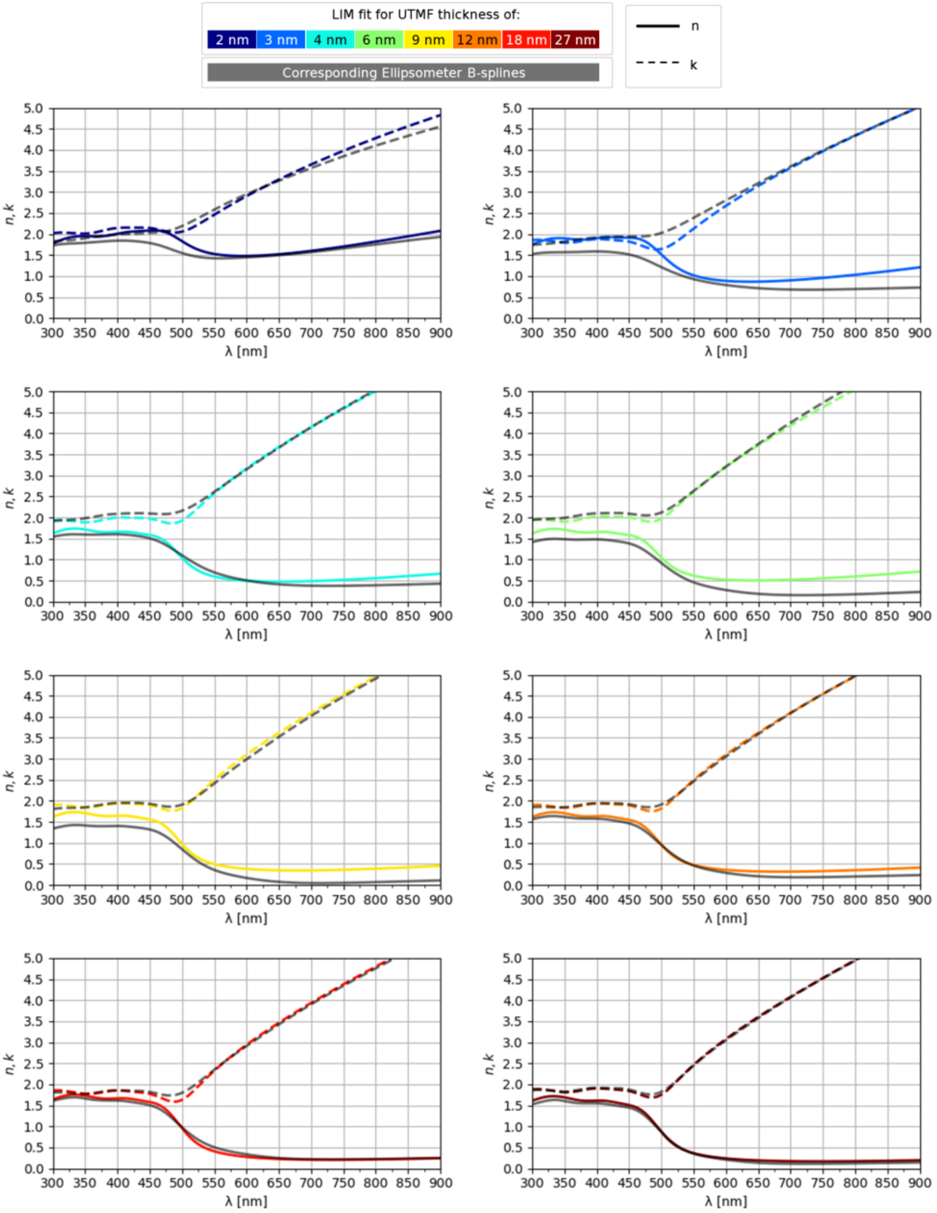

Fig. S4: Comparison of modelled complex RIs for LIM-fit and for ellipsometer B-spline fit, with varying thickness from 2 to 27 nm

SI-5. COMPARISON OF REFLECTANCE MEASURED BY SPECTROMETER AND SIMULATION BASED ON FITTED PARAMETERS

As an additional verification of the optical model and complex refractive indices, we simulate the expected reflectance spectra for the UTMF of varying thicknesses and compare them with reflectance measurements from a commercial spectrometer (Perkin Elmer LAMBDA 950), which are independent datasets that were not used for the fitting. Therefore, if the spectrometer reflectance matches the simulations, this corroborates the fitted model and complex refractive indices. Fig. S3 shows this comparison, and the simulated and measured reflectance spectra are in fact in good agreement. Therefore, a transmission only phase and intensity measurement can also be used to determine the reflectance of a thin film through the modelling process described in our work.

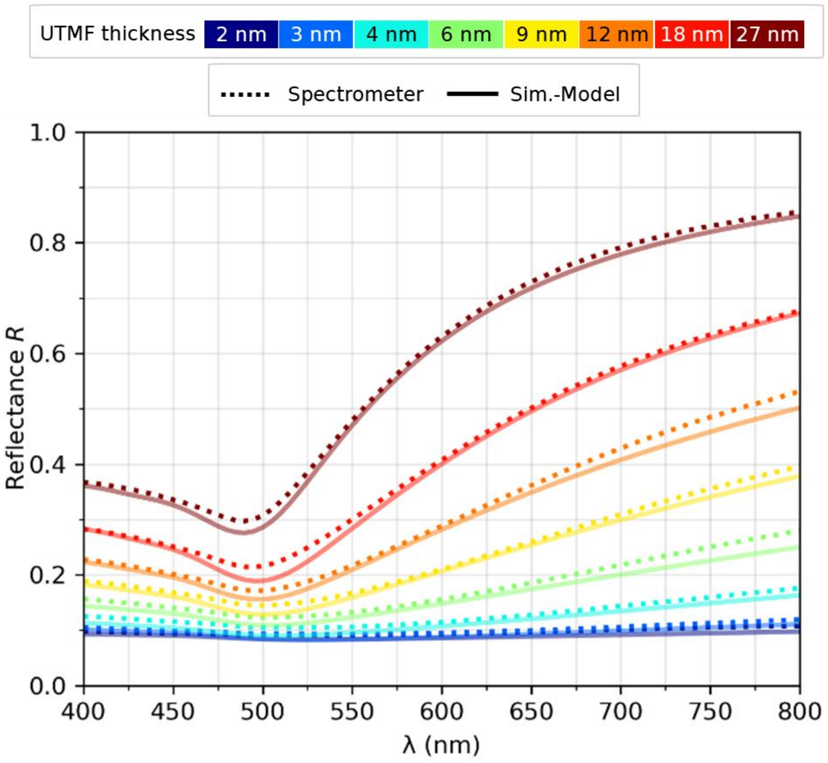

Fig. S5: Comparison of measured reflectance and simulated reflectance based on the proposed optical model and determined complex refractive indices

SI-6. INFLUENCE OF CUO SEED LAYER ON SIMULATED *T, OPD, R*

We take the optical model and the determined $\tilde{n}$ (see main document) and simulate the Au UTMF's optical response when including the seed CuO layer. To model the CuO layer, we assume a layer thickness $d_{CuO} = 0.5$ nm and using $\tilde{n}$ of bulk CuO taken from the material database of our ellipsometer (J.A. Woollam VASE) – see Fig. S4 bottom-right. The resulting spectral values for *T*, *OPD* and *R* are displayed in Fig. S6. As it can be seen, the contribution of seed CuO layer is tiny and can be considered negligible.

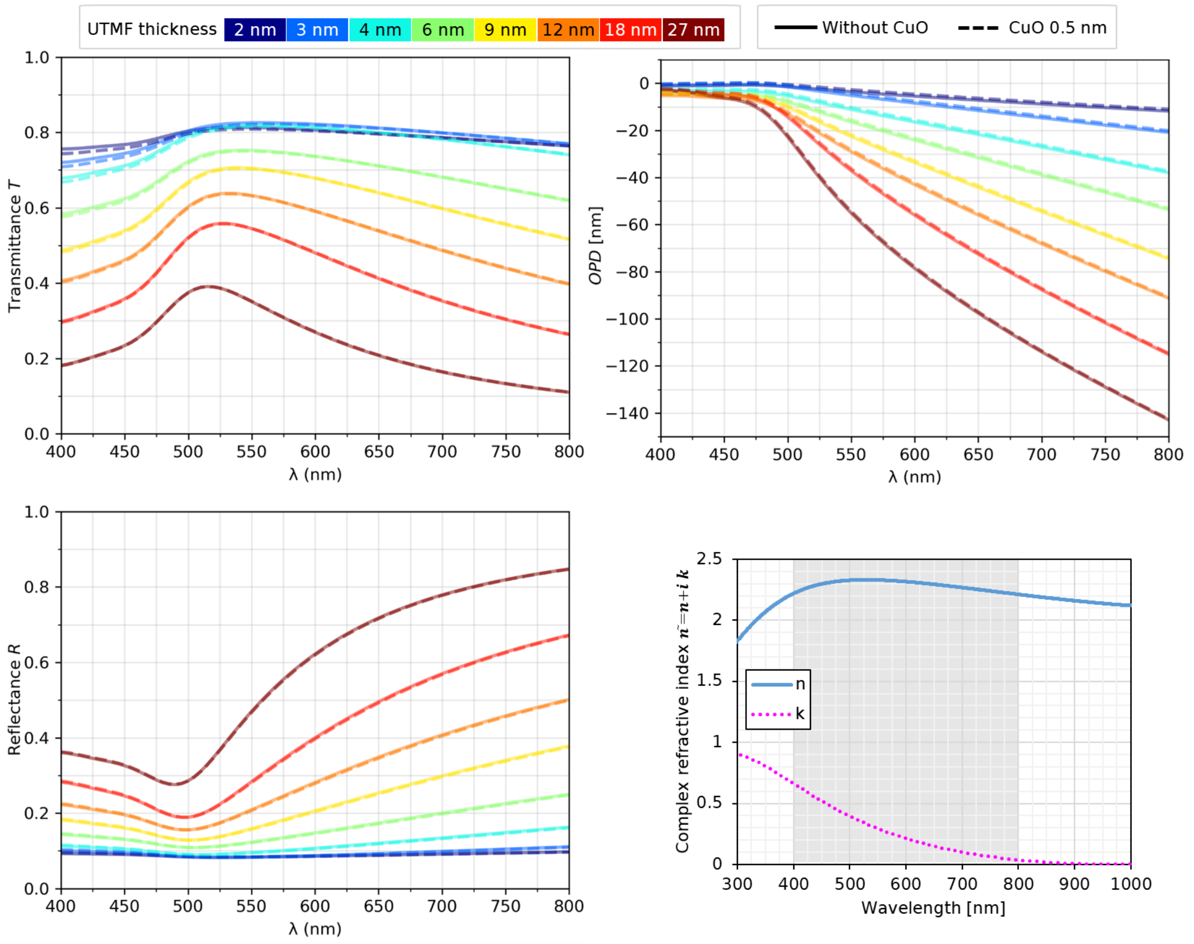

Fig. S6: Simulations for T, OPD and reflectance R for varying UTMF thickness with (dashed line) and without (solid line) 0.5 nm seed CuO layer. The bottom-right graph shows $\tilde{n} = n + i \cdot k$ for CuO used in the simulation.

SI-7. REFERENCES:

(1) Martínez-Cercós, D.; Paulillo, B.; Maniyara, R. A.; Rezikyan, A.; Bhattacharyya, I.; Mazumder, P.; Pruneri, V. Ultrathin Metals on a Transparent Seed and Application to Infrared Reflectors. *ACS Appl. Mater. Interfaces* **2021**, *13* (39), 46990–46997. https://doi.org/10.1021/acsami.1c10824.

(2) Mendoza-Herrera, L. J.; Tebaldi, M. C.; Scaffardi, L. B.; Schinca, D. C. Determination of Thickness-Dependent Damping Constant and Plasma Frequency for Ultrathin Ag and Au Films: Nanoscale Dielectric Function. *Phys. Chem. Chem. Phys.* **2022**, *24* (45), 28019–28028. https://doi.org/10.1039/D2CP04286J.

(3) Yakubovsky, D. I.; Arsenin, A. V.; Stebunov, Y. V.; Fedyanin, D. Y.; Volkov, V. S. Optical Constants and Structural Properties of Thin Gold Films. *Opt. Express* **2017**, *25* (21), 25574. https://doi.org/10.1364/OE.25.025574.

(4) Yakubovsky, D. I.; Stebunov, Y. V.; Kirtaev, R. V.; Ermolaev, G. A.; Mironov, M. S.; Novikov, S. M.; Arsenin, A. V.; Volkov, V. S. Ultrathin and Ultrasmooth Gold Films on Monolayer MoS 2. *Adv. Mater. Interfaces* **2019**, *6* (13), 2–7. https://doi.org/10.1002/admi.201900196.

(5) Olmon, R. L.; Slovick, B.; Johnson, T. W.; Shelton, D.; Oh, S.-H.; Boreman, G. D.; Raschke, M. B. Optical Dielectric Function of Gold. *Phys. Rev. B* **2012**, *86* (23), 235147. https://doi.org/10.1103/PhysRevB.86.235147.
